# CohortDiagnostics: Phenotype evaluation across a network of observational data sources using population-level characterization

**DOI:** 10.1371/journal.pone.0310634

**Published:** 2025-01-16

**Authors:** Gowtham A. Rao, Azza Shoaibi, Rupa Makadia, Jill Hardin, Joel Swerdel, James Weaver, Erica A. Voss, Mitchell M. Conover, Stephen Fortin, Anthony G. Sena, Chris Knoll, Nigel Hughes, James P. Gilbert, Clair Blacketer, Alan Andryc, Frank DeFalco, Anthony Molinaro, Jenna Reps, Martijn J. Schuemie, Patrick B. Ryan

**Affiliations:** 1 Observational Health Data Analytics, Janssen Research and Development, LLC, Titusville, NJ, United States of America; 2 OHDSI Collaborators, Observational Health Data Sciences and Informatics (OHDSI), New York, NY, United States of America; 3 Department of Biostatistics, University of California, Los Angeles, CA, United States of America; 4 Department of Biomedical Informatics, Columbia University, New York, NY, United States of America; University of Siena: Universita degli Studi di Siena, ITALY

## Abstract

**Objective:**

This paper introduces a novel framework for evaluating phenotype algorithms (PAs) using the open-source tool, Cohort Diagnostics.

**Materials and methods:**

The method is based on several diagnostic criteria to evaluate a patient cohort returned by a PA. Diagnostics include estimates of incidence rate, index date entry code breakdown, and prevalence of all observed clinical events prior to, on, and after index date. We test our framework by evaluating one PA for systemic lupus erythematosus (SLE) and two PAs for Alzheimer’s disease (AD) across 10 different observational data sources.

**Results:**

By utilizing CohortDiagnostics, we found that the population-level characteristics of individuals in the cohort of SLE closely matched the disease’s anticipated clinical profile. Specifically, the incidence rate of SLE was consistently higher in occurrence among females. Moreover, expected clinical events like laboratory tests, treatments, and repeated diagnoses were also observed. For AD, although one PA identified considerably fewer patients, absence of notable differences in clinical characteristics between the two cohorts suggested similar specificity.

**Discussion:**

We provide a practical and data-driven approach to evaluate PAs, using two clinical diseases as examples, across a network of OMOP data sources. Cohort Diagnostics can ensure the subjects identified by a specific PA align with those intended for inclusion in a research study.

**Conclusion:**

Diagnostics based on large-scale population-level characterization can offer insights into the misclassification errors of PAs.

## Introduction

Phenotype algorithms (PA) are computerized queries used to identify specific clinical events or conditions in health data sources, such as electronic health records or administrative claims [1–4]. PA are foundational elements in almost every real-world analysis. Misclassification errors in a PA, may threaten the reliability of evidence generated from observational studies [5]. Identifying and remediating such misclassification errors are challenging [6, 7].

Conceptually, the process of designing PA for a study includes two main steps: phenotype development and phenotype evaluation. Several techniques have been developed to help researchers author algorithms to identify patients with a specific phenotype [6, 7]. These tools facilitate tasks related to developing PAs, such as searching codes and constructing logic with Boolean and temporal operators. While such tools are essential, techniques for efficiently evaluating the performance of the resulting PAs by assessing different types of misclassification errors are still scarce.

Misclassification errors can be assessed using metrics such as sensitivity, specificity, positive predictive value (PPV), and negative predictive value (NPV). These metrics depend on comparison to a gold standard reference classifier, such as a comprehensive disease registry or medical record reviews. Unfortunately, disease registries are not always available, and even when they are, they often cover only a limited range of conditions and might be incomplete [8]. Medical record reviews, while valuable, are resource-intensive, time-consuming, prone to interobserver bias, and unfeasible in large deidentified data sources [9, 10]. Furthermore, most medical record reviews provide only PPV information.

Recent advances have led to the introduction of scalable alternatives to chart reviews such as CALIBER and PheValuator [11, 12]. Although these novel methods report on the existence and magnitude of measurement errors, they do not identify the sources of these errors or suggest modifications to the PA to enhance its performance. Prior publications on phenotyping have unscored the need for reproducible framework to systematically evaluate PA’s for the detection, quantification, and reduction of misclassification errors [2, 13].

In this paper we propose a framework to address these gaps, introducing a framework for phenotype evaluation as a separate but iterative step to phenotype development. This framework supplements existing methods for PA evaluation and can be embedded into an iterative process of PA development and evaluation.

In this work, we aim to introduce a new framework for phenotype evaluation by assessing potential misclassification errors in PAs using population-level characterization. This framework has been integrated into CohortDiagnostics, an open-source software that is able to run on person level health data in Observational Medical Outcomes Partnership (OMOP) common data model format [14]. To illustrate its effectiveness, we apply this methodology to two distinct health conditions represented as computable phenotypes: Systemic Lupus Erythematosus (SLE) and Alzheimer’s Disease (AD).

## Materials and methods

### Overview

**Fig 1:**
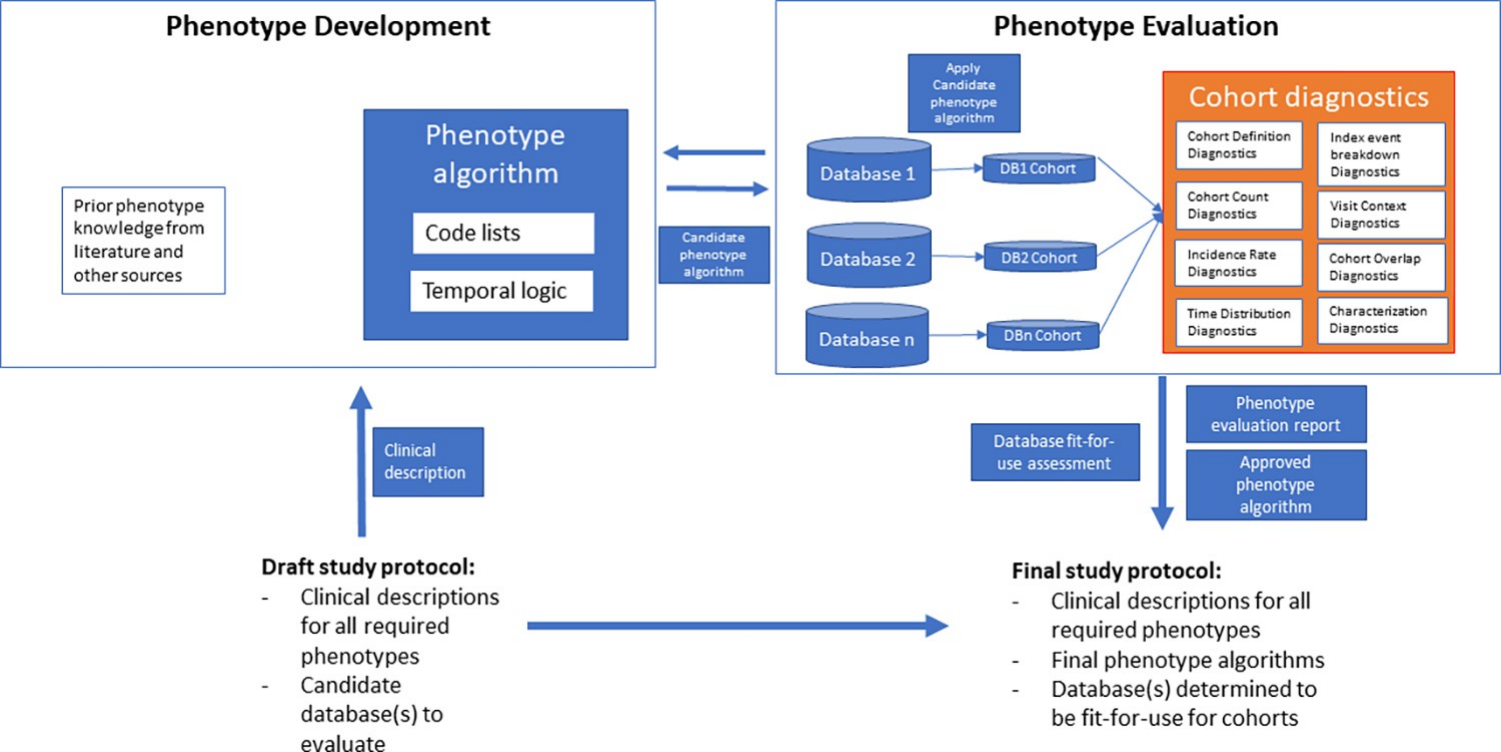
Conceptual process of phenotype development and evaluation, highlighting where the proposed evaluation framework fits within the full process of designing an observational study.

Fig 1 illustrates the conceptual process of phenotype development and evaluation, highlighting where our proposed evaluation framework fits within the full process of designing an observational study. As shown in the figure, an initial draft of a study protocol specifies all required study phenotypes that need to be identified in the data (with an unambiguous clinical description). This clinical description serves as the input for the phenotype development step, which leads to candidate phenotype algorithms that then need to be evaluated. We conceptualize an evaluation step that includes implementing the candidate PA on the candidate data source(s), identifying potential misclassification errors that can be possibly eliminated by modifying the candidate PA, and finally feeding back into the final study protocol. This process outputs an approved PA, a phenotype evaluation report, and a database fit-for-use assessment.

We propose a data-driven approach to evaluate PAs (the orange inner box), based on a set of summary statistics (characterization of the cohort) that serve as diagnostic indicators. Each of these diagnostics provides insights into potential misclassification errors. To clarify, when a PA is run against a data source, the result is a ‘cohort’. A cohort is a set of individuals who satisfy all the criteria specified in the PA for a duration of time represented by cohort_start_date and cohort_end_date. Cohort_start_date is the calendar date in a data source corresponding to the entry criteria of the PA that meets all other inclusion criteria. Cohort_end_date is the calendar date in a data source corresponding to the exist criteria specified in the PA. For example, a PA for diabetes can have an entry criterion of a diagnosis of diabetes and an exit criterion of the end of continuous observation in the data source. Additional terms are described as glossary in supplementary material S1 File. ‘CohortDiagnostics’ is an open-source software tool that generates and visualizes summary statistics called diagnostics. These diagnostics include estimates of incidence rate (see details in the next section); the breakdown of entry event codes on the index date (i.e., cohort entry); the distribution of type of visits prior to, on, and after the index date; and the prevalence of all observed clinical events prior to, on, and after the index date. Table 1 lists the entire set of diagnostics that are available in CohortDiagnostics and provides a guide on how to use it.

**Table 1:** Diagnostics and Guide on using diagnostics to infer misclassification error.

**Cohort Definition Diagnostics**
Review the codes by vocabulary that are part of the PA, such as International Classification of Diseases (ICD), SNOMED-CT
Check if semantically appropriate codes have been selected as part of the PA
Examine if any codes in the resolved code set are semantically inconsistent with the clinical description of the target phenotype
Identify any missing or orphaned codes based on PHOEBE (PHenotype Observed Entity Baseline Endorser) [15, 16]
**Cohort Count Diagnostics**
Check if PAs produce a cohort with zero counts in one or more data sources
Evaluate if the same PA produces lower than anticipated cohort counts in one data source, compared to other data sources
Look for substantial differences in counts for similar PAs when applied to the same data source
If the PA allows a person to enter the cohort at multiple distinct temporal periods, examine the ratio of subject count to event count
Identify any inclusion rules that have no subjects or very few subjects satisfying in a data source
Find any inclusion rules that drastically reduce the cohort counts in a data source
Evaluate how the impact of inclusion rules differs across data sources
**Incidence Rate Diagnostics**
Check if the incidence rate is monotonous and stable when stratified by calendar year
Look for any sudden or abrupt change in pattern, and investigate possible explanations. Note: strata with low denominator count needs to be removed from consideration to avoid statistical instability.
Evaluate the consistency of the incidence rate across demographic sub-groups
Examine if the incidence rate stratified by age*sex*calendar year follows an explainable pattern
Compare incidence rates with those reported from external sources
Check for any strata that appear lower compared to the other strata, e.g., < 100 subjects
**Time Distribution Diagnostics**
Check if the people in the cohort are observed in the data source for less than expected duration for any of the three time distributions (Time in days between a person’s observation start date in the data source and their cohort start date; time in days between a person’s cohort start date and cohort end date; Time in days between a person’s cohort start date and the last date of continuous observation in the data source)
Look for persons with low or even 0 days observed in the three time distributions.
Evaluate if the time distribution follows a uniform or skewed distribution
Compare the time distributions across data sources
**Index Event Breakdown Diagnostics**
Identify the main concept(s) that are driving entry into cohort
Evaluate the consistency of the concepts driving entry of subjects into the cohort across data sources
Check if cohort entry is predominantly or unexpectedly due to a certain code
**Visit Context Diagnostics**
Determine the most common type of visit that a person in the cohort experiences around cohort entry
Check if the visit type associated with the cohort entry event matches expectations
Evaluate the utilization rates of Emergency department or Inpatient hospitalization shortly before, during or after index
**Cohort Overlap Diagnostics**
Determine the proportion of subjects present in both PAs compared to only one, when overlapping two similar PAs for the same phenotype.
**Characterization Diagnostics**
Check for concepts that should be present but aren’t (e.g., proportions of common treatments, known diagnostic procedures, known risk factors or coexisting conditions).
Identify concepts that shouldn’t be seen but are (e.g., Contradictory condition).
Look for other concepts that may suggest presence of disease that may invalidate the phenotype
Examine concepts in the time prior that suggest the outcome started earlier (e.g., Specific treatment, cooccurring conditions, specific diagnostic work, a complication or an exacerbation of the condition, the concepts in the concept set expression)?
Compare cohort characteristics for the same PAs when observed over multiple data sources

#### Incidence rates

For each PA the Incidence rates are calculated for all permutations of 10-year age groups, sex, and calendar year strata in each data source. These rates were computed by dividing the number of identified cases of the PA (i.e., the number of subjects meeting each Phenotype Algorithm criteria) by the total person-time at risk. The person-time at risk is the sum of person-years contributed by all eligible individuals within a data source (i.e. the patient’s population in a data source). Rates are expressed per 1000 person-years. The incidence rates are provided for all years of available data. By exploring the estimated incidence rate a reviewer can 1. cross-reference the observed rate with expected or known epidemiological trends reported in the existing literature (e.g. known incidence of a disease in a general population) 2. evaluate the stability of the IR overtime. Typically, incidence rates stratified by calendar year are anticipated to be present in a continuous, unvarying monotonous temporal pattern with no abrupt shifts. Should any interruptions in this pattern be observed, it could suggest alterations in either clinical practices or data capture processes, leading to potential inconsistencies in PA performance. 3. to explore whether the distribution of age and gender aligns with the known epidemiological trends for each phenotype. Additionally, this diagnostic approach can be utilized to ascertain if any data source exhibited an IR inconsistent with others, suggesting potential issues with specific definitions within that data source. On the other hand, consistent incidence estimate across multiple data sources can suggest positive reliability of the PA.

#### Index event breakdown

Index event breakdown shows the count of cohort entries where a specific code in the PA’s entry event criteria, coincided with the index date of cohort entry. In other words, these are codes that likely triggered the cohort entry. The frequency of these codes allows us to assess their individual contributions to the cohort.

If most individuals are entering the cohort based on a limited number of the total specified codes in the PA, this could potentially point towards specificity errors. A higher occurrence of codes that might be semantically narrower compared to the clinical definition of the phenotype may suggest sensitivity errors. Additionally, any variations in the rank order of codes among different data sources might indicate measurement heterogeneity.

#### Visit context

This diagnostic presents the count of individuals who experienced different types of healthcare visits (outpatient, inpatient, emergency department) in relation to the index date, as follows: 1) ‘Before’ represents visits that concluded within 30 days prior to the index date. 2) ’During’ accounts for visits that began before and extended up to or beyond the index date. 3) ’Simultaneous’ covers visits that initiated on the index date. 4) ’After’ includes visits that commenced within 30 days post the index date.

We anticipate that certain types of visits will be more common for specific patient phenotypes. For instance, severe acute conditions requiring intensive care will probably result in inpatient visits. A preponderance of unanticipated visit types might suggest a specificity error.

#### Cohort overlap

The cohort overlap diagnostics conducts pairwise comparison of cohorts from two PAs, reporting the individuals identified by either one or both PAs, as well as only one PA. This diagnostics in CohortDiagnostics is visualized using a Venn diagram or a table. Examining the overlap between two different PAs representing the same disease can provide insights into the potential sensitivity loss associated with one algorithm compared to the other.

#### Cohort characterization

Cohort characterization diagnostics provides a overview of the cohort using descriptive statistics on demographic factors, condition, drug exposures, measurements, and occurrences of procedure codes. For each selected data source, CohortDiagnostics displays the prevalence of all observed clinical events (denoted by codes) at different time periods relative to the index date. Default time windows include a) 365 to 31 days prior to the index date, b) 30 to 1 day before the index date, c) on the index date, d) 1 to 30 days post the index date, and e) 31 to 365 days post the index date.

Clinical events are represented through one or more codes. The prevalence is given for each code individually, and some are grouped using a vocabulary hierarchy. This diagnostic feature allows us to simulate, at the cohort level, the process by which clinicians establish and confirm clinical diagnoses. We expect individuals diagnosed with a certain disease to exhibit its signs and symptoms on or before the index date. Similarly, we anticipate diagnostic tests related to the disease to occur on or before its onset, followed by relevant treatment occurrences on or after onset. A lack of such expected characteristics might point to misclassification errors. To enable comparative analyses across multiple PAs, the tool carries out pairwise comparisons of all observed characteristics for each assessed PA. The results, including proportions or means and the standardized (mean) difference for each covariate, are presented in tables and scatter plots.

*Application*. Our evaluation of PAs focuses on two distinct scenarios: First, a researcher may examine a single PA on its own merit, by looking for possible misclassification errors across one or more data sources. This evaluation could guide the researcher in adjusting the PA in successive iterations to reduce possible misclassification errors. Second, a researcher may compare the diagnostic performance of two or more PAs that represent the same clinical concept in the same data source. This would help the researcher infer which PA might has lower misclassification errors, allowing them to choose from the two the PA that offers the best performance.

To illustrate these two scenarios, we implemented our proposed framework on two clinical concepts of interest—SLE and AD. Before evaluating the PA, we ensure we understand the known clinical profile of persons we are attempting to capture in the data source. This is done by writing a clinical description for medical condition/disease, with elements like overview, presentation, diagnostic evaluation, therapy plan, risk factors, and prognosis. The authored clinical description serves as a tool that enables documentation of the shared understanding among researchers of the target clinical idea. It also provides justification for the phenotype development design choices and expected clinical attributes to look for during phenotype evaluation.

#### Phenotypes

*System Lupus Erythematous*. SLE is an autoimmune disease with a wide range of severity characterized by periods of exacerbation and relative quiescence and occurs predominantly among women of child-bearing age (15 to 44 years). Based on SLE clinical description, we developed a PA that allows patients to enter the cohort on the earliest of either a diagnosis code, treatment for (ie, hydroxychloroquine, steroids, biologics, or immunosuppressants) or signs and symptoms related to SLE (ie, Inflammatory dermatosis, rash, joint or back pain, endocarditis), as long as there was at least one diagnosis code for SLE within 0 to 90 days from the entry date. All patients were required to have at least 365 days of continuous observation prior to the index date. The full PA for SLE including condition and drug codes and temporal logic is in supplementary material S2 File. The validity of this PA to identify patients with SLE against a diagnostic predictive model, which serves as the gold standard, was estimated in a prior study utilizing the same data sources used in this paper [17]. Sensitivity ranged between 0.64 and 0.98 and PPV ranged from 0.244 to 0.74.

*Alzheimer’s disease*. AD is an age associated progressive neurodegenerative disorder and the most common cause of dementia [18]. For AD, we constructed 2 PAs. The first AD PA (referred to as the simple PA) allows patients to enter the cohort on first occurrence of an AD diagnosis. Prior studies estimated that the sensitivity of Medicare claims to identify Alzheimer’s disease using an AD diagnosis was 64.2%, and the positive predictive value (PPV) was 58.3% [19]. The second AD cohort is a more restrictive and is derived from the work by Imfeld et. al., [20] requiring one of 3 inclusion criteria: 1) the first occurrence of AD diagnosis as the entry event criterion, and any of the following inclusion criteria in relation to entry date: a) a prescription on or after for AD drug, b) a second AD diagnosis any time after, c) a prior dementia test, d) a prior, simultaneous, or subsequent dementia symptom, or e) if the first occurrence was diagnosed in an inpatient setting; or having the 2) first occurrence of dementia followed by at least 2 prescriptions for AD drugs; or 3) prescription for AD drugs followed by a diagnosis of AD. Individuals were excluded if they were under 18 years of age at cohort start date, were subsequently diagnosed with diseases that, when present, make the diagnosis of AD less likely (e.g., Vascular dementia, Lewy Body disease, Pick’s disease), or had an occurrence of a stroke diagnosis within 2 years before index date. In their study, Imfeld et.al. reports that this PA was validated through questionnaire to GPs which confirmed AD diagnosis for 79% of the AD cases.

*Datasources*. The data sources used in the evaluation are described in supplementary material S1 Table. We included 6 claims based data; JMDC, Merative^TM^ MarketScan® Commercial Claims and Encounters Database (CCAE), Merative^TM^ MarketScan® Medicare Supplemental and Coordination of Benefits Database (MDCR), Merative^TM^ MarketScan® Multi-State Medicaid Database (MDCD), IQVIA® Adjudicated Health Plan Claims Data (Pharmetrics Plus), Optum’s Clinformatics® Data Mart—Socio-Economic Status (Optum SES) and 4 electronic medical record (EHR) data; IQVIA® LPD in Australia (LPDAU), IQVIA® Disease Analyzer France (France DA), IQVIA® Disease Analyzer Germany (German DA), Optum® de-identified Electronic Health Record dataset (Optum EHR). These data sources have been de-identified at source and the involved researchers did not have information to re-identify the data. The data has been standardized to the Observational Medical Outcomes Partnership (OMOP) Common Data Model (CDM) [21, 22]. Extract, transform, and load (ETL) specifications for all data sources except LPDAU, France DA, German DA, and Pharmetrics Plus are available at ETL-LambdaBuilder [23]. The standardized data are assessed using a rigorous data quality process to evaluate conformance, completeness, and plausibility of the data [24]. For this application, we utilized the entirety of the data available without calendar time restrictions or sampling. The calendar time covered in each data source varied; supplementary material S1 Table lists the earliest and latest calendar times for each data source. Subjects were identified as patients with SLE or AD according to the phenotype algorithms detailed in the previous section. The six data sources selected for this application provide diversity in patient demographics, geographic locations, and healthcare settings. This diversity is crucial for evaluating the consistency of our phenotype algorithms across different data sources.

*Data analysis (CohortDiagnostics software)*. CohortDiagnostics is open-source software application written in the R programming language that implements the described theoretical framework [25]. Given a set of instantiated cohorts, a set of cohort definition details, and a connection to a remote database with person level data converted to the OMOP CDM [21, 22] (version 5.3+), CohortDiagnostics produces a set of aggregate summary statistics called Diagnostics. The output contains no patient-level data and has additional privacy protection using minimum cell count thresholds [26]. All output conforms to the prespecified CohortDiagnostics results data model and is formatted as unencrypted comma separated value (.csv) files (an intentional design decision to allow an investigator to audit compliance with privacy governance). The output.csv files, from one or more data sources, may then be combined and the results reviewed using an interactive R Shiny web application called DiagnosticsExplorer. The software and user documentation are available on OHDSI Github repository called CohortDiagnostics [27].

## Results

Table 2 summarizes the number of patients who met the definitions for SLE and the 2 AD PAs in each data source.

**Table 2:** Cohort counts for the phenotype algorithms for Systemic Lupus Erythematous and Alzheimer disease.

	SLE	Alzheimer’s disease		
Data Source	Count	Count: Simple	Count: Imfeld, 2013	Relative difference (Imfeld/simple)
CCA	435,810	38,413	24,073	37.3%
France DA	223	3,804	718	81.1%
German DA	10,776	106,663	26,963	74.7%
JMDC	31,600	9,064	3,554	60.8%
LPDAU	673	1,052	373	64.5%
MDCD	99,165	349,543	111,224	68.2%
MDCR	53,697	479,742	290,711	39.4%
Optum EHR	260,614	540,074	435,949	19.3%
Optum SES	348,541	840,314	430,062	48.8%
Pharmetrics Plus	375,000	288,092	137,527	52.3%

SLE = Systemic Lupus Erythematous

Imfeld, P., et al, Seizures in patients with Alzheimer’s disease or vascular dementia: a population-based nested case-control analysis. Epilepsia, 2013. 54(4): p. 700–7

Below we provide brief overviews of the key insights informed by the evaluation process. The full output of CohortDiagnostics is available in the interactive website [28].

### Systemic Lupus Erythematous

**Fig 2:**
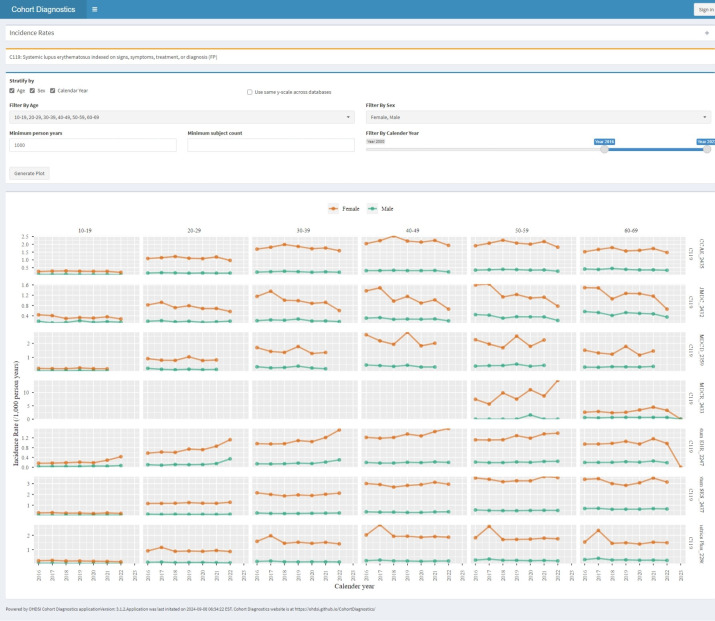
Incidence rate of Systemic Lupus Erythematous stratified by age decile, gender, and calendar year.

*Insights from incidence rate plots*. Fig 2 illustrates the pattern of incidence rate of SLE in each data source, stratified by age, gender and calendar year. Except for two general practitioner data sources France Disease Analyzer (France DA) and Germany Disease Analyzer (Germany DA), we observed high concordance across data sources for an incidence range from 0.51 to 1.24 per 1000 person-years. Incidence rate estimation variation due to database heterogeneity can be substantial, and such variation is not necessarily an evidence of measurement error [29]. However, observing concordance among data sources provides some reassurance that the PA measurement error is not causing substantial incidence rate heterogeneity. In this case, observing that the incidence rates in the Germany DA and France DA data sources are substantially different from those in other data sources may suggest that the PA is possibly not suitable for use in these two data sources.

As expected, females have approximately 5-fold greater incidence of SLE compared with males. However, the rates increase by age, and peak around 40 to 50 years, which is slightly older than previously reported typical age of SLE onset of 15–44 years [30]. This may imply sensitivity error among younger age patients (eg, younger women may receive treatment for SLE like symptoms without a diagnosis) or index date misclassification (eg, older patients may already have had the disease but its onset was not recorded in the data source).

*Insights from index event breakdown*. Across data sources, a substantial proportion of individuals enter the SLE cohort based on SLE symptoms or treatment. This indicates that many patients receive treatment for SLE, before their diagnosis is coded and recorded for administrative or clinical purposes. That is, a diagnosis date is observed in the data source, but this date lags the date persons could be presumed to have the disease (represented by date treatment or symptom onset). This represents index date misclassification error.

Lastly, all the events (appearing as codes) observed on the index date are related to SLE, which suggests the absence of specificity error or false positives.

**Fig 3:**
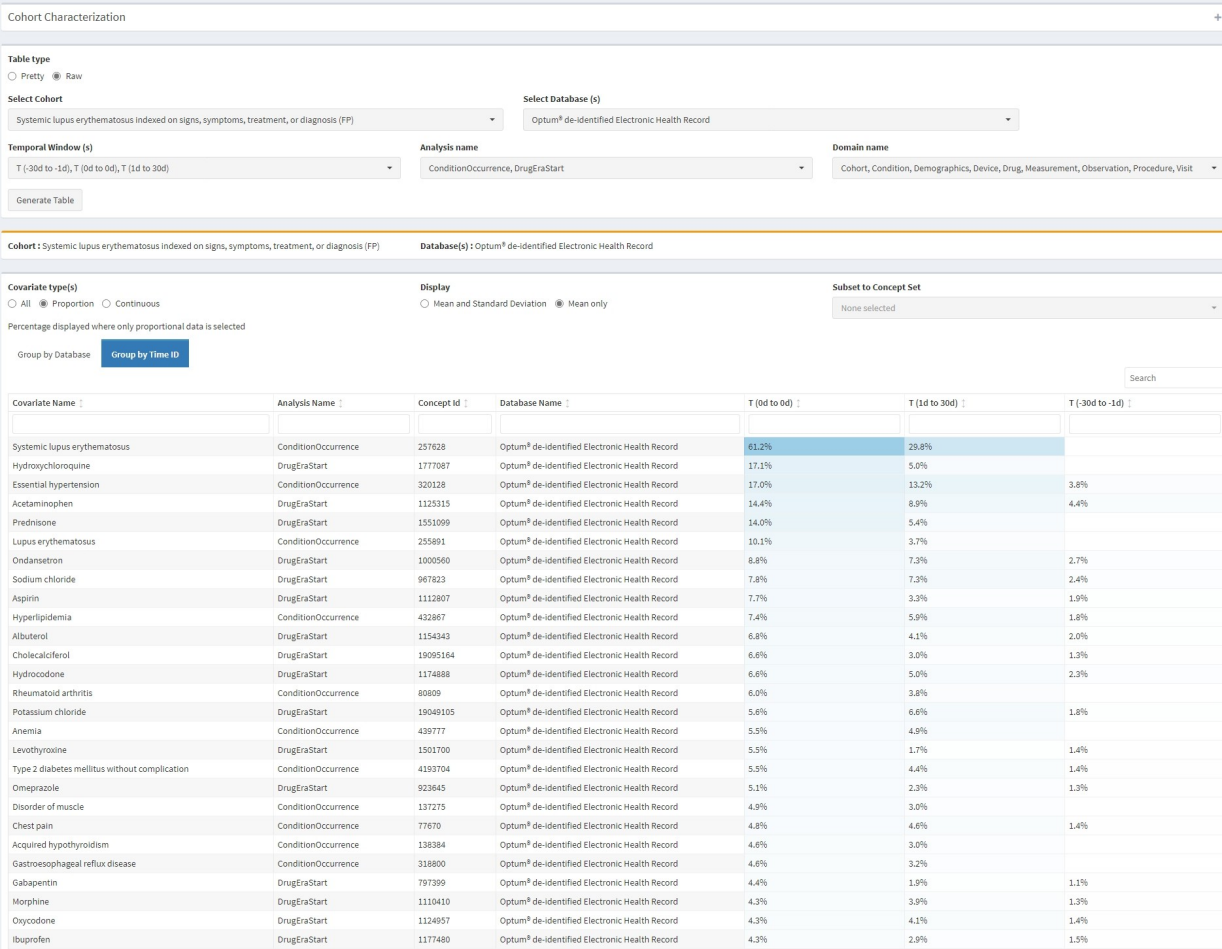
Characterization output from CohortDiagnostics tool showing the most prevalent conditions and drug exposures on or around index date.

*Insights from cohort characterization*. Fig 3 is a screen shot from the CohortDiagnostics tool showing the most prevalent conditions and drug exposures observed in the Optum® EHR data source among the SLE cohort on the index date. SLE treatments such as prednisone, hydroxychloroquine, methotrexate and cyclophosphamide were observed on or shortly after the index date. Some individuals started these drugs in the period 365 to 30 day prior to index date, indicating potential index date misclassification. However, since the definition index on signs, symptoms and other allied diagnoses, no other related conditions are frequently observed prior index. Consistent with the clinical description of SLE which stated that follow-up visits were expected, we observed SLE diagnosis codes occurring post index (30–50%). Laboratory tests such as urinalysis and antinuclear antibody were also observed (e.g., in 7 to 10% in Optum® EHR on index date) and these tests clustered temporally around the index date. Observing expected baseline and post index characteristics and clinical events suggests that the patients returned by the SLE PA are likely true cases and that misclassification may be limited.

#### Alzheimer disease

*Insights from cohort overlap*. In all data sources, the Imfeld et.al. PA returned fewer patients (19% to 81%) compared with the simpler AD PA (Table 1). [20] In the cohort overlap, among individuals who were present in either cohort, the proportion of individuals present in both cohorts ranged between 18% and 45%. Further, 35% to 81% of individuals were identified only by the simple PA; and 0% to 20% were identified only by the Imfeld et al PA. We can infer that the simpler PA is likely to have higher sensitivity compared with the Imfeld et al PA.

*Insights from visit context*. The distributions of the visit type around the index date among the 2 cohorts were comparable in most data sources with less than 10% of the individuals in either cohorts identified during or at the start of an inpatient visit. This suggests that neither AD PAs were likely to capture more AD during an acute care event.

*Insights from cohort characterization*. Table 3 reports a selected set of characteristics from the 2 AD cohorts at baseline i.e., from 365 days before the index date up to and including the index date in the Optum® EHR data source (Data from all other data sources are available in the CohortDiagnostics shiny app). The covariates are defined using the Systematized Nomenclature of Medicine Clinical Terms (SNOMED-CT) vocabulary hierarchy grouping. We observe that even though the 2 cohorts were defined using different PAs and have considerably different number of patients with less than 50% overlap, the distributions of the main baseline characteristics were comparable.

**Table 3:** Selected baseline characteristics among patients with Alzheimer’s disease by phenotype algorithm in Optum® EHR.

Characteristic	Simple (n = 540,074)	Imfeld et al (n = 435,949)	Characteristic	Simple (n = 540,074)	Imfeld et al (n = 435,949)
**Age group**			**Cardiovascular disease**		
55–59	1%	1%	Atrial fibrillation	14%	13%
60–64	2%	3%	Cerebrovascular disease	8%	5%
65–69	5%	5%	Coronary arteriosclerosis	15%	14%
70–74	11%	10%	Heart disease	36%	33%
75–79	32%	33%	Heart failure	12%	10%
80–84	37%	36%	Ischemic heart disease	7%	6%
85–89	11%	10%	Peripheral vascular disease	5%	4%
**Gender**			Pulmonary embolism	1%	1%
FEMALE	64%	63%	Venous thrombosis	1%	1%
MALE	36%	37%	**Neoplasms**		
**Race**			Hematologic neoplasm	1%	1%
Black or African American	8%	8%	Malignant neoplastic disease	6%	6%
White	79%	84%	Malignant tumor of breast	1%	2%
**General**			Primary malignant neoplasm of prostate	1%	1%
Acute respiratory disease	7%	6%			
Chronic liver disease		1%			
Chronic obstructive lung disease	9%	9%			
Dementia	100%	84%			
Depressive disorder	16%	15%			
Diabetes mellitus	18%	18%			
Gastroesophageal reflux disease	12%	12%			
Gastrointestinal hemorrhage	2%	2%			
Hyperlipidemia	30%	30%			
Hypertensive disorder	47%	44%			
Obesity	3%	4%			
Osteoarthritis	13%	13%			
Pneumonia	6%	6%			
Renal impairment	18%	16%			
Rheumatoid arthritis	1%	1%			
Schizophrenia	1%				
Urinary tract infectious disease	13%	11%			
Visual system disorder	8%	7%			

**Fig 4:**
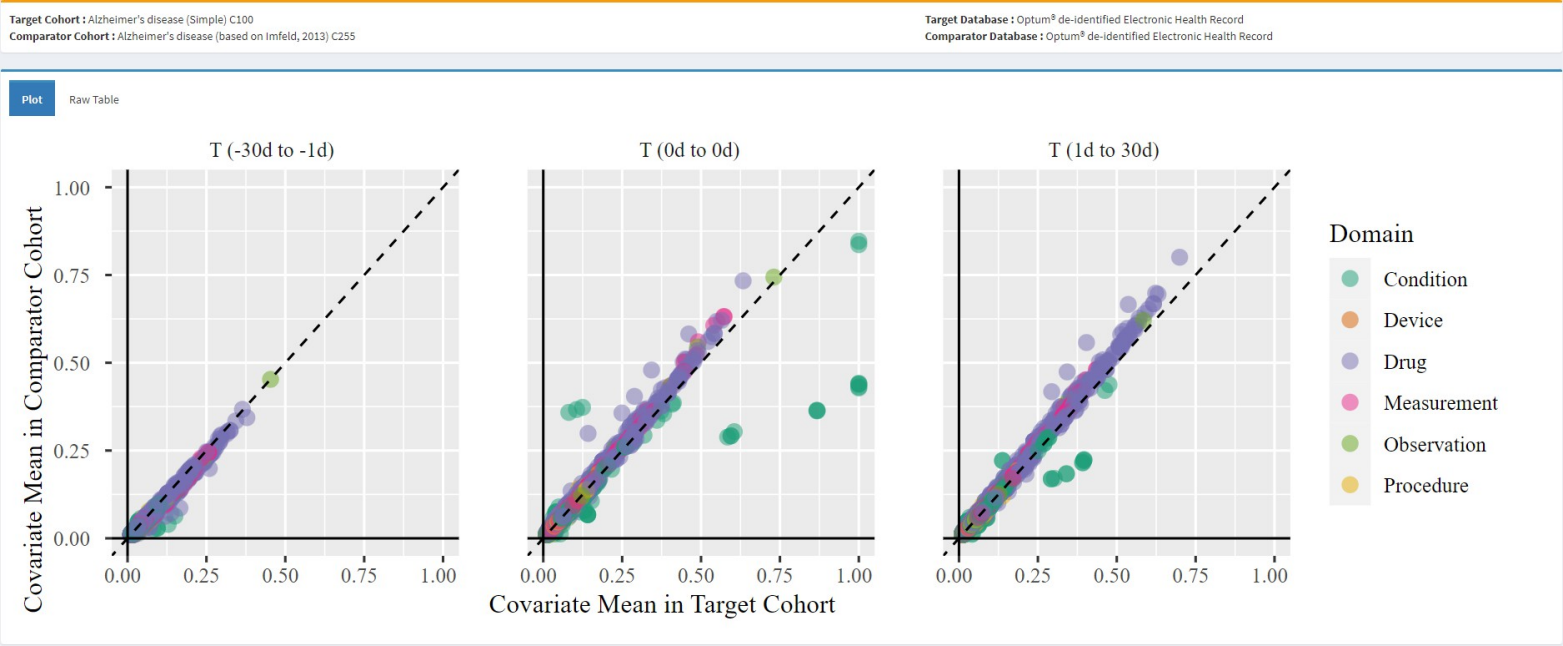
Covariate balance between the 2 Alzheimer’s disease phenotype algorithms.

Fig 4 is a screenshot from CohortDiagnostics that illustrates the covariate balance between the 2 AD PAs in Optum® EHR on 3 different time periods around the index date. Overall, we observed that most features near the diagonal, indicating comparable cohort characteristics distribution between the 2 cohorts. However, some covariates are off the diagonal with a larger Standardized Mean Difference. For example, during 30 days to 1 days before index, we observed higher prevalence of vascular dementia, and other late effects of cerebrovascular accidents in the simple PA compared with the Imfeld et al PA. This suggests that the Imfeld et al PA is less likely to misclassify cerebrovascular accident events as AD. We also observed that the simpler PA had higher utilization of drugs commonly used in AD such as donepezil and memantine in the same immediate period prior to index date. Conversely, the Imfeld et al PA demonstrated higher utilization of these drugs on index. Both PAs had similar utilization after index. This suggests that the simple PA is subject to higher index date misclassification compared with the complex PA.

When we compared covariates constructed from codes that were not part of either AD PA entry event, we observed considerable cohort similarity. This suggests that the 2 PAs identified patients with similar clinical profiles despite incomplete cohort overlap. Overall, the descriptive data of these PAs for AD revealed that, while the Imfeld et. al. identified fewer patients (raising concerns about its sensitivity), we did not observe a higher prevalence of clinical characteristics that strongly suggest a higher specificity when compared with the other simple PA.

## Discussion

We have developed and integrated an empirical methodology for the evaluation of PAs into a new tool designed for the OMOP Common Data Model called CohortDiagnostics. We have demonstrated this evaluation framework on one SLE and 2 AD PAs. Our evaluation framework categorizes errors into three types: sensitivity errors, specificity errors and index date misclassification errors, providing a consistent means of assessment. This approach allows for the identification and assessment of these errors in any PA by reviewing population-level characterization.

In our application, we conclude that the SLE PA demonstrates acceptable operating characteristics and is suitable for use across most of the data sources assessed, despite the potential index event misclassification. However, the PA might not be suitable for data from general practitioners, such as France DA and Germany DA. On the other hand, we found that the Imfeld et al PA may have lower sensitivity than the simple PA. The simple PA has index event misclassification and a potential specificity error explained by the observed cerebrovascular accidents events. Additionally, our data suggest that the sensitivity/PPV tradeoff between the two PA varies across the data sources.

These findings align with and build upon what is already known about the validity of the three PAs from prior studies when compared to a gold standard. For SLE, our conclusion is consistent with the findings of Swerdel et al. [17], who reported an acceptable PPV and sensitivity for the SLE PA across US data sources but a poorer performance in the Germany DA (PPV of 0.244). For AD, Imfeld et al. [20] reported acceptable specificity but did not address the sensitivity of the algorithm. Other validation studies have reported on the trade-off between sensitivity and PPV when using alternative AD algorithms [31]. However, most of these studies were conducted within the context of one or two data sources, while our study explores these trade-offs across a network of data sources.

We have shown that this empirical and scalable framework for PA evaluation offers insights into misclassification errors. It not only detects the existence of these errors but provides an understanding of their direction and magnitude. We demonstrate that it can provide reasons for the origin of such errors, enabling researchers to refine their PAs iteratively. This method can work together with traditional case-level retrospective medical record adjudication or innovative approaches like PheValuator, which quantify estimates of measurement error [12, 17]. When paired with validation analyses for quantifying measurement errors, our population-level characterization leads to a comprehensive understanding of a PA’s performance.

Our software tool, CohortDiagnostics, performs extensive diagnostics across multiple data sources for one or more PAs. It presents results in a privacy-compliant format. It is designed to perform phenotype evaluation across an observational database network. This feature allows a coordinator site to distribute a self-contained phenotype evaluation study package to each contributing data partner site, which can then independently execute it. After the execution, each site may share aggregate summary statistics back to the coordinator site, complying with local data governance and privacy policies. These site-level summary statistics can then be aggregated into one integrated viewer for collaborative review. This aggregated data can be used by a team of experts to discuss the merits of the PAs under evaluation and to understand associated misclassification errors. This framework has been recently implemented in numerous observational network studies and collaborations [32–35]. Notably, the Data Analysis and Real-World Interrogation Network (DARWIN EU®)has recently outlined a 14-step reproducible framework for reliable and traceable phenotype generation that incorporate CohortDiagnostics as the main tool to perform diagnostic check and evaluate disease phenotypes for observation research [36].

The network-based phenotype evaluation process reinforces confidence in a PA. It allows for the evaluation of the consistency of diagnostics across different data sources, geographical locations, and time periods. Consistent trends in misclassification errors increase our confidence that our PAs have reliable operating characteristics, rather than representing an artifact from a specific data source. Such findings are crucial as they support the conclusion that a PA is applicable across various data sources. Moreover, evaluating a PA across a network offers valuable insights into different clinical settings, practices, and data capture processes. We are optimistic that this framework will encourage the use of more robust and externally valid PAs.

CohortDiagnostics also informs code selection during phenotype development. Selecting the right set of code to represent a clinical idea of interest is known be challenging and inconsistent [37]. While code selection should be guided by clinical judgment, the empirical impact of these judgment can be readily evaluated through our tool. This evaluation can measure the effect of alternative codes on the PA performance by assessing the impact on counts and characteristics.

Despite its strengths, our approach has some limitations. It cannot numerically quantify measurement errors and should be used in conjunction with other methods that include a gold standard, such as PheValuator, or other validation methods [38]. Furthermore, analyzing descriptive results to gain insights on misclassification errors can be subjective and time-consuming. More methodological research is required to formalize a scalable, reproducible process and establish empirically driven. Finally, this approach is based on the assumption that the evaluation data sources have been standardized to the OMOP CDM and have undergone data quality review and it is fit for research use [24].

In this paper, we introduce a framework for phenotype evaluation, that is intended to be done as part of designing observational research. It helps ensure that the individuals identified by the PA are consistent with the profiles of the patients we intend to study in a particular data source. Utilization of this framework enhances researchers’ confidence in the validity of their study outcomes. The framework has been integrated into the CohortDiagnostics software. We have shown how this open-source software can enable collaborative research within a broad research community and can scale to multiple PAs, over multiple data sources that can be repeated over multiple time periods enabling creation of a repository of such evaluations [3, 4, 36].

## Supporting information

S1 File Glossary of terms S1 File. (DOCX)

S2 File Description of phenotype algorithm. (DOCX)

S1 Table Description of data sources. (XLSX)
