# Supplementary material for: CohortDiagnostics: Phenotype evaluation across a network of observational data sources using population-level characterization
Source: PLoS One. 2025 Jan 16;20(1):e0310634. doi: 10.1371/journal.pone.0310634 (PMC11737733; doi:10.1371/journal.pone.0310634)
Supplement: S1 File — (DOCX) [file pone.0310634.s001.docx]

**Glossary of terms:**

**Cohort Entry Events:** These are specific clinical events or conditions that qualify a person to enter a cohort. They mark the starting point for an individual's inclusion in the cohort. Entry events can be diagnoses, procedures, drug exposures, or any other identifiable event in the data.

**Cohort Exit:** This defines the conditions or events that mark the end of an individual's time in a cohort. The exit can be based on a fixed period, the occurrence of another clinical event, the end of data availability, or other specified criteria.

**Cohort Eras:** These are continuous time periods during which an individual is considered to be a part of a cohort. A cohort era starts with a cohort entry event and continues until a cohort exit event. Multiple eras can occur for a single individual if they meet the entry and exit criteria multiple times.

**Concept Sets:** These are groups of concepts that define clinical conditions, drugs, procedures, or other health-related phenomena. Concept sets are used to specify the criteria for cohort entry and exit events. They consist of standard and non-standard concepts from various vocabularies.

**Concept ID:** A unique identifier assigned to each concept within the OHDSI standardized vocabulary. Concept IDs are used to reference specific clinical terms, drugs, procedures, or other items in a consistent manner across different data sources.

**Concept Name:** The descriptive name associated with a Concept ID. It provides a human-readable label for the concept, such as "Diabetes mellitus" or "Aspirin 500mg oral tablet."

**Code:** A specific identifier from an external vocabulary or coding system (e.g., ICD-10, SNOMED CT, RxNorm) that represents a clinical term, drug, procedure, or other items. Codes are mapped to standard concepts within the OHDSI vocabulary.

**Mapped Concepts:** These are non-standard concepts from various coding systems that are mapped to standard concepts within the OHDSI standardized vocabulary. This mapping ensures consistency and interoperability across different data sources and vocabularies.
